# The combined ADHD profile faces the greatest risk of delay, depletion and disengagement in university students

**DOI:** 10.1038/s41598-026-41256-1

**Published:** 2026-02-24

**Authors:** Vanessa Müller, Bettina Pikó

**Affiliations:** 1 Doctoral School of Education, University of Szeged, Szeged, Hungary; 2 MTA–SZTE Digital Learning Technologies Research Group, University of Szeged, Szeged, Hungary; 3 Department of Behavioral Sciences, University of Szeged, Szeged, Hungary

**Keywords:** ADHD, university students, procrastination, perfectionism, ego depletion, dropout intention, Psychology, Risk factors, Quality of life, Comorbidities, Fatigue, Psychiatric disorders, ADHD

## Abstract

Attention-deficit/hyperactivity disorder (ADHD) symptoms are increasingly recognized in adulthood, particularly among university students, yet their distinct psychological correlates remain underexplored. Using a cross-sectional design, this study examined Hungarian college students (*N* = 1,879, aged between 18 and 35 years) who responded online to a set of questionnaires that included the Adult ADHD Self-Report Scale, Procrastination Scale, Higher Education Retention Scale and Ego-Depletion Scale. We applied k-means cluster analysis to classify students into four ADHD-related groups (Combined, Inattentive, Hyperactive-Impulsive, Low-symptom) based on symptom criteria. Consequently, we compared these clusters across six theoretically relevant self-regulatory outcomes: maladaptive and adaptive procrastination, maladaptive and adaptive perfectionism, ego depletion and dropout intention. A multivariate analysis of covariance (MANCOVA), controlling for age and biological sex, revealed significant differences among ADHD clusters on all outcomes. While the Combined group reported the highest levels of maladaptive procrastination and ego depletion, the Hyperactive-Impulsive and Low-symptom groups reported the highest adaptive procrastination scores. These findings suggest that adaptive procrastination may serve as a protective or compensatory strategy in students with certain ADHD profiles, offering constructive reconceptualization of functional delay and self-regulation in academic contexts.

## Introduction

Attention-Deficit/Hyperactivity Disorder (ADHD) has traditionally been examined through the lens of early childhood, yet a growing body of evidence indicates that its core symptoms –particularly inattention, impulsivity, and poor self-regulation – persist into adulthood for a substantial portion of individuals^1^. In the context of higher education, this continuity holds significant implications. University settings demand sustained mental effort, time management, emotional control, and self-initiated learning – domains that map directly onto the core difficulties of ADHD. For many students, this results in not merely academic underperformance but a broader pattern of psychological strain and functional vulnerability^2^.

A global meta-analysis by Song et al.^3^ reported that persistent adult ADHD affects 2.58% and symptomatic adult ADHD affects 6.76% of the general adult population. In contrast, a study by Mak et al.^4^ found that approximately 15.9% of university students exhibit ADHD symptoms. In Hungary, this discrepancy may be further amplified by systemic underdiagnosis and limited access to assessment, leaving many students unsupported despite clinically significant symptom profiles^5^. Conceptually, adult ADHD is increasingly understood as a self-regulatory condition in which impairments in inhibitory control and state regulation translate into functional costs^6^, particularly under sustained academic demands. In higher education, these costs are often expressed in recurring patterns of task delay in this population^7^, self-evaluative strain^8^, and reduced persistence, making procrastination and perfectionism theoretically relevant behavioral correlates^9,10^. Ego depletion offers a complementary lens on the subjective burden of prolonged self-control and has been linked to ADHD symptoms^11^ and procrastination-relevant self-regulatory breakdowns in student populations^12^. Finally, dropout intention captures a proximal marker of academic disengagement and has demonstrated predictive value for subsequent attrition^13^.

Although some studies have documented academic outcomes such as lower GPA (Grade Point Average) or increased dropout risk in students with ADHD^14^, less is known about the intermediate psychological markers – the patterns and connection of procrastination, perfectionism, and ego depletion – that may help explain how ADHD manifests functionally in academic life.

### Dropout Intention as an Early Warning Sign for Attrition in ADHD

Students with ADHD face significantly higher college attrition rates than their non-ADHD peers. For example, one longitudinal study found only about 49% of unmedicated undergraduates with ADHD persisted through 4 years, compared to 59% of students without ADHD^9^. Research in educational settings shows a positive link between expressed intent to drop out and actual dropout behavior^13^. In fact, knowing a student’s dropout intentions can serve as an “early alert signal” of eventual withdrawal^15^. Taken together, these findings suggest that when a university student with ADHD symptoms reports thinking about dropping out, it should be taken seriously as an indicator of heightened attrition risk. As a proximal predictor of attrition, it offers a measurable, timely indicator of academic risk – one that can be addressed before irreversible decisions are made. Incorporating this variable into contemporary research allows for earlier identification of students in distress and supports the development of targeted interventions aimed at retention.

### Active and passive procrastination

Primarily, procrastination has been considered as a negative, dysfunctional behavior, characterized by voluntarily delay in an action leading to negative consequences, such as failure in keeping deadlines^16^. Academic procrastination can lead to academic failure, cheating, or even dropout^17^. However, several studies differentiate between active and passive procrastination: while the passive form might be viewed as undesirable aspect of procrastination, the active form could be a functional/purposeful delay with an intention to achieve the best outcomes^18^. Maladaptive (passive) procrastination often reflects a breakdown of executive functioning – particularly the capacity to initiate tasks under internal control^19^. In contrast, adaptive procrastination has been described as a strategic behavior, where intentional delay is used as a motivator under time pressure^20^. Active procrastination is a complex construct that involves a combination of satisfaction with results, intentional delays in task completion, an eagerness to meet deadlines, and a tendency to thrive under pressure^18^. Those who engage in active procrastination often perform better academically^10^ and show enhanced abilities in creative thinking^21^. Procrastination, particularly its maladaptive (passive) form, is among the most frequently reported functional difficulties in students with ADHD^22^ and is typically characterized by disorganized task initiation and chronic delay despite adverse consequences^18^. Research suggests that individuals with ADHD are more prone to the maladaptive subtype, possibly due to weaker self-monitoring and greater distractibility^23^. Adaptive forms of procrastination may offer temporary relief or performance advantages in some students, but whether students with ADHD can engage in such strategies remains empirically unclear.

### Maladaptive and active perfectionism

Perfectionism, a personality trait with high standard setting and concerns with mistakes and negative evaluations of others^24^, is often linked to academic achievement^25^. Similarly to procrastination, perfectionism presents in both adaptive and maladaptive forms, with the latter being linked to distress, avoidance, and lower academic satisfaction^26^. Maladaptive perfectionism is marked by an ongoing effort to meet unrealistically high standards, coupled with a tendency toward harsh self-criticism. Maladaptive perfectionism may be particularly problematic for students with ADHD, who report high self-criticism despite struggling to meet their own standards^10^. In contrast, adaptive perfectionism is characterized by the pursuit of high personal standards, accompanied by strong organizational skills and effective planning^27^. It seems to possess a set of additional skills that enable them to navigate academic expectations more effectively. The relationship between adaptive perfectionism and psychological well-being appears to be inconclusive in literature. While some studies suggest a positive link^28^, others have failed to provide evidence supporting such a connection^26^. Whether adaptive perfectionism acts as a helpful or protective factor for students with ADHD symptoms remains uncertain at this point, as the potential benefits or drawbacks of such tendencies have yet to be clearly established.

### Ego depletion

Finally, the concept of ‘ego depletion’ refers to the prominent model that aims to explain its effects – the strength model of self-control^27^. According to this model, self-control depends on a finite, domain-independent resource that becomes partially depleted after any act of self-control, making an individual more susceptible to failure in subsequent self-control efforts. At the cognitive level, this model highlights the depletion of a mental or physical resource as the key mechanism driving the behavioral effects of ego depletion^28^. Ego depletion has also been linked to increased procrastination, as reduced self-control undermines task initiation and persistence^29^. Students with ADHD may experience more rapid or more frequent depletion, given the cognitive load imposed by sustained self-regulation. When depleted, these students may become more vulnerable to disengagement, impulsivity and emotional exhaustion – factors that are rarely included in formal academic assessments but play a leading role in the lived experience of educational stress^30^. Yet few studies have empirically examined how specific ADHD profiles relate to clusters of behavioral vulnerability within student populations.

### Differences across age and biological sex

Recent findings also suggest that both age and sex may moderate the expression and impact of ADHD symptoms in academic contexts^31^. Neurodevelopmentally, the transition from adolescence into emerging adulthood (typically defined as ages 18–29)^32^ is marked by continued maturation of executive functioning and emotional regulation systems, particularly in the prefrontal cortex (PFC), posterior parietal cortex, and superior temporal cortex^33^. These processes may interact with ADHD symptoms, influencing how self-regulatory difficulties manifest during university life. Similarly, research has shown that sex differences are relevant across multiple domains of ADHD expression: females with ADHD often present with higher internalizing symptoms and perfectionistic tendencies, while males more commonly show externalizing behaviors and impulsivity^34^. Procrastination and ego depletion also appear to differ modestly across sex, with females often reporting higher academic stress and males’ higher behavioral disengagement^35^. Accordingly, age and sex were treated as key sources of heterogeneity in the present study, in addition to the focal study variables.

### The current study

The present study investigates the above mentioned associations in a large Hungarian sample of university students. The core of our interest is to test whether ADHD symptom profiles differ meaningfully on six outcomes: maladaptive and adaptive procrastination, maladaptive and adaptive perfectionism, dropout intention and ego depletion. Each of these constructs has been separately linked to ADHD in the literature, yet their joint distribution across symptom profiles stays unexplored, especially in non-Anglophone academic settings. Using a four-cluster model of ADHD symptom expression – Combined type, Inattentive type, Hyperactive-Impulsive type, and a Low/no-symptom comparison group – we aim to investigate their associations with three psychological dimensions central to academic adjustment. This approach is intended not only to clarify the psychological sequela of ADHD in academic contexts but also to find potential subgroups at heightened risk for academic stress and dysfunction. Considering the findings regarding age and sex, these variables were both included as covariates in the current study, and participants were descriptively grouped into emerging adults and young adults based on developmental stage. Emerging adulthood is defined broadly as ages 18–29^32^, operational cut-offs can vary across contexts. Although all participants were enrolled as students, we used 25 years as a pragmatic threshold to distinguish the modal university-age cohort from mature-age students, who are more likely to have non-linear study trajectories and additional work or caregiving roles^36^. In doing so, the study contributes to a more differentiated understanding of ADHD in higher education: not simply as a monolithic disorder of inattention or hyperactivity, but as a set of self-regulatory vulnerabilities that manifest in specific, measurable behavioral patterns. By attending to procrastination style, perfectionistic tendencies, dropout intention, and the subjective experience of depletion, this research offers a framework for interpreting ADHD beyond symptoms alone – focusing on how these symptoms translate into psychological risk in the everyday context of university life.

## Methods

### Participants

A cross-sectional study was conducted between January and April 2024 among Hungarian university students. Data were gathered via a self-administered online questionnaire hosted on the Typeform platform. Participants were eligible if they: (a) were over 18 years old; (b) were currently enrolled at a Hungarian university; (c) provided written informed consent; (d) reported no difficulty understanding the questions; and (e) confirmed that they had responded truthfully and attentively. No participants were excluded based on these criteria. Participants were between 18 and 35 years old (M = 22.31, SD = 3.60 years) and enrolled in Hungarian institutions of higher education. The final sample included 1,879 students, of whom 70% identified as female (*n* = 1,316) and 30% as male (*n* = 563). Most were full-time students (88.3%, *n* = 1,660), with smaller proportions in part-time (11%, *n* = 207) or distance-learning programs (0.6%, *n* = 12). Regarding educational level, 64.3% (*n* = 1,208) were bachelor’s students, 8.8% (*n* = 166) were enrolled in master’s programs, 19.9% (*n* = 373) in undivided master’s programs, 56 (3%) in short-cycle higher education program and 4% (*n* = 76) in doctoral training. Respondents first provided demographic data, then completed the psychological scales, and 68 participants (3.6%) reported a previous diagnosis of ADHD. All materials were administered in Hungarian, and the dataset contained no missing responses. All participants identified Hungarian as their primary language.

### Measurement tools

#### ADHD symptoms scale

ADHD symptoms were assessed using the Adult ADHD Self-Report Scale (ASRS v1.1), developed by the World Health Organization^37^. The ASRS consists of 18 self-report items (e.g., “How often do you fidget or squirm with your hands or feet when you have to sit down for a long time?”), rated on a five-point Likert scale from 0 (never) to 4 (very often). The instrument is designed as a screening tool to identify individuals who may require further clinical evaluation for ADHD. It is not intended to establish a diagnosis. Total scores range from 0 to 72, with higher scores indicating greater symptom severity. Consistent with prior research^38,39^, the current study used the total ASRS score as a continuous measure of ADHD symptomatology. Internal consistency for the scale in this sample was high (Cronbach’s α = 0.84).

#### Procrastination scale

Active and passive procrastination were measured using the Hungarian eight-item version of the Procrastination Scale^40^, a validated short form of the original Active–Passive Procrastination Questionnaire^11^. The scale comprises two subscales: four items assess passive procrastination (e.g., “Even after I make a decision, I delay acting upon it”), and four items assess active procrastination (e.g., “I finish most of my assignments right before deadlines because I choose to do so”). Participants rated each item on a seven-point Likert scale ranging from 1 (not typical at all) to 7 (totally typical). Subscale scores were calculated as the mean of the respective four items, with higher scores indicating greater levels of the given procrastination type. Internal consistency in the present sample was acceptable (Cronbach’s α = 0.78).

#### Dropout intention scale

Dropout intention was measured using the Higher Education Retention Questionnaire^41^. The full Hungarian version of the instrument includes 17 items across six subscales: support from teachers (e.g., “I felt supported by my teachers”), expectations of one’s own performance (e.g., “I strived to go to my exams as prepared as possible”), transparency of expectations (e.g., “It was clear to me what I had to do to complete my coursework”), social involvement (e.g., “I felt I didn’t belong to any social group at university”), intention to drop out (e.g., “I was thinking about suspending my studies”), and academic/study involvement (e.g., “I liked my studies”). Items are rated on a six-point Likert scale ranging from 1 (almost never) to 6 (almost always). In this study, only the “intention to drop out” subscale was used. Internal consistency of this subscale was excellent in the original validation (α = 0.94), and similarly high in the present sample (Cronbach’s α = 0.90).

#### Perfectionism Scale

Perfectionism was measured using the Frost Multidimensional Perfectionism Scale – Brief (FMPS-B), which contains eight items divided into two subscales: evaluative concerns (maladaptive) and strivings (adaptive), each comprising four items. Responses are rated on a five-point Likert scale ranging from 1 (strongly disagree) to 5 (strongly agree), yielding subscale scores from 4 to 20 and a total score range of 8 to 40. Higher scores reflect stronger perfectionistic tendencies. The Hungarian version of the scale has been previously adapted and applied in student populations^42^. In the present study, the scale demonstrated good internal consistency (Cronbach’s α = 0.81).

#### Ego Depletion Scale

To assess ego depletion sensitivity, we employed the Ego Depletion Questionnaire developed by Salmon et al.^43^, which consists of 11 items measuring individual differences in susceptibility to mental fatigue (e.g., “After I have made a couple of difficult decisions, I can be truly mentally “depleted”). Responses were rated on a 7-point Likert scale ranging from 1 (totally disagree) to 7 (totally agree). High scores on these items are expected to reflect heightened sensitivity to depletion. The scale demonstrated good internal consistency in the current sample (Cronbach’s α = .84).

### Data analysis

All analyses were conducted using IBM SPSS 26.0 Statistics (IBM, Chicago, IL). Descriptive statistics were first calculated to examine the distributional properties of the study variables and to screen for missing data, outliers, or violations of parametric assumptions. No variable exceeded acceptable thresholds for skewness or kurtosis |2.0|, and all scales proved acceptable internal consistency.

To explore symptom-based variation in psychological risk profiles, participants were grouped into four ADHD symptom profiles using k-means clustering based on z-standardized scores for inattention and hyperactivity-impulsivity. A four-cluster solution was selected a priori based on theoretical consistency with DSM-5^44^ ADHD presentation types. Iterations terminated after 10 steps, with a maximum final cluster center shift of 0.022. The resulting groups reflected: (1) Combined (high inattention and hyperactivity), (2) Inattentive (high inattention, low hyperactivity), (3) Hyperactive-Impulsive (high hyperactivity, low inattention), and (4) Low-symptom (low on both dimensions). Although ANOVA results confirmed significant between-cluster differences on the input variables, these comparisons were descriptive only, as the clustering algorithm is designed to maximize separation along the selected features. As such, the clusters are treated as exploratory, theory-guided groupings.

Group differences across six outcomes – maladaptive procrastination, adaptive procrastination, maladaptive perfectionism, adaptive perfectionism, dropout intention and ego depletion – were tested using a multivariate analysis of covariance (MANCOVA), with age and sex included as covariates. These variables were selected due to prior evidence suggesting developmental and sex-related differences in ADHD symptom expression and self-regulatory functioning during university years. For descriptive purposes, participants were also grouped into two age categories: emerging adults (18–24 years) and young adults (25 + years), consistent with developmental frameworks. Given concerns about normality and the robustness of parametric inference in large samples, a bias-corrected and accelerated (BCa) bootstrap procedure with 5,000 resamples was applied. The Mersenne Twister algorithm was used as the random seed generator (seed = 2025) to ensure reproducibility. Pairwise post hoc comparisons were adjusted using Bonferroni correction, and 95% confidence intervals were reported for all effects.

Effect sizes were interpreted using partial eta squared (η²p), with values of 0.01, 0.06, and 0.14 considered small, medium, and large, respectively^45^. A significance level of *p* < .05 was used throughout. Z-scores were used exclusively for the clustering procedure. All subsequent analyses, including the MANCOVA and pairwise comparisons, were conducted using raw (unstandardized) scale scores. As all data in this study were collected through self-report measures, which may introduce common method bias, Harman’s single-factor test was used to evaluate the extent of this potential bias. The results indicated that the first factor accounted for 19.82% of the variance, well below the critical threshold of 40%^46^. This suggests that common method bias is not a significant concern in this study. Finally, analyses to determine the minimum sample size for MANCOVA were conducted by using G*Power 3.1.9.7.

### Ethics and consent declarations

The study was conducted according to the guidelines of the Declaration of Helsinki and approved by was approved by the Institutional Review Board of the Doctoral School of Education, University of Szeged (reference number: 21/2023). Informed consent was obtained from all participants prior to data collection.

## Results

### Descriptive statistics and bivariate associations

Prior to the MANCOVA analyses, we examined the intercorrelations between all the measured variables (see Table 1). The results showed that dropout intention was significantly and positively associated with inattention symptoms, hyperactivity-impulsivity symptoms, maladaptive procrastination, maladaptive perfectionism, and ego depletion. Adaptive procrastination and adaptive perfectionism were also significantly correlated with dropout scores, though in opposite directions. Among self-regulatory measures, maladaptive and adaptive perfectionism were strongly intercorrelated, suggesting they may co-occur in individuals despite theoretical differences. Ego depletion was positively related to all ADHD symptoms and maladaptive behaviors and showed a particularly strong correlation with inattention. Inattention and hyperactivity were moderately correlated, indicating shared but distinct variance. The skewness and kurtosis values of the measured variables were within the range of ± 2.0, indicating that the distribution did not deviate significantly from normality^47^. All correlated variables were included in further analyses.

**Table 1:** Descriptive Statistics and Pearson’s Correlations for Study Variables for the Full Sample (*N* = 1879).

Variable	M ± SD	Skewness (Kurtosis)	Min. (Max.)	1	2	3	4	5	6	7
(1) Hyperactivity	16.53 ± 6.08	0.32 (-0.06)	1 (36)	–						
(2) Inattention	17.94 ± 6.35	0.23 (-0.27)	1 (36)	0.53^**^	–					
(3) Maladaptive procrastination	18.06 ± 6.12	-0.33 (-0.77)	4 (28)	0.26^**^	0.57**	–				
(4) Adaptive procrastination	15.86 ± 5.14	0.08 (-0.58)	4 (28)	− 0.08^**^	− 0.25**	− 0.18**	–			
(5) Maladaptive perfectionism	12.59 ± 3.79	-0.01 (-0.66)	4 (20)	0.26**	0.14**	0.07**	− 0.15**	–		
(6) Adaptive perfectionism	12.39 ± 3.83	0.01 (-0.70)	4 (20)	0.27**	0.14**	0.05*	− 0.14**	0.94**	–	
(7) Ego depletion	51.44 ± 11.37	-0.178 (-0.29)	20 (77)	0.39**	0.64**	0.48**	− 0.35**	0.21**	0.22**	–
(8) Dropout intention	6.85 ± 4.20	1,01 (-0.09)	3 (18)	0.18**	0.34^**^	0.24**	− 0.20**	0.15**	0.13**	0.36**

Note. * *p <* .05, **p < .001

### Assumption Checks and Multivariate Effects

Prior to conducting the main analyses, we also examined key assumptions. Box’s M test was significant (*p* < .001), suggesting heterogeneity of covariance matrices. Accordingly, Pillai’s Trace was used for multivariate effects as it is the most robust to this violation^48^. Levene’s tests showed unequal error variances for two outcomes – maladaptive procrastination, *F*(3, 1875) = 16.62, *p* < .001, and adaptive procrastination, *F*(3, 1875) = 2.68, *p* = .046 – suggesting some heteroscedasticity. To address this, we employed a bootstrapped MANCOVA (5,000 samples, Mersenne Twister seed = 2025) and report bootstrapped confidence intervals (BCa, 95%), which are robust to non-normality and heteroscedasticity^49^.

The omnibus MANCOVA revealed a significant multivariate main effect of ADHD cluster on the combined dependent variables (Pillai’s Trace = 0.399, *p* < .001, partial η² = 0.133, observed power = 1.00). This says that the four ADHD groups (Combined type, Inattentive type, Hyperactive-Impulsive type, Low-symptom group) differ significantly on the overall profile of procrastination, perfectionism, ego depletion, and dropout intention measures. There was no multivariate effect of age group (Pillai’s Trace = 0.001, *F*(6, 1873) = 0.44, *p* = .851, partial η² < 0.001), nor was there a significant cluster × age group interaction. In contrast, a significant multivariate effect of biological sex appeared (Pillai’s Trace = 0.072, *F*(6, 1873) = 24.17, *p* < .001, partial η² = 0.072), indicating small overall differences between male and female students across the six dependent variables.

### Univariate ANCOVA Results

Follow-up univariate ANCOVAs (with age and biological sex as covariates) showed that ADHD cluster had a significant effect on each of the six dependent variables (Table 2). All outcome measures differed across the four ADHD groups at the *p* < .001 level.

**Table 2:** Descriptive Statistics (Means and Standard Deviations) and Group Differences for Each Dependent Variable by ADHD Cluster (*N* = 1,879).

Variable	Combined *(n = 339)*	*Inattentive (n = 550)*		Hyperactive-Impulsive *(n = 471)*		Low-symptom *(n = 519)*		F		*p*		η²*p*	
Maladaptive procrastination	22.20 (4.61)	20.24 (5.08)		16.13 (5.80)		14.80 (5.84)		179.66		< 0.001		0.223	
Adaptive procrastination	14.60 (5.30)	14.96 (4.79)		16.58 (5.21)		16.99 (5.00)		24.46		< 0.001		0.038	
Maladaptive perfectionism	14.17 (3.83)	12.19 (3.64)		12.94 (3.77)		11.69 (3.59)		33.35		< 0.001		0.051	
Adaptive perfectionism	13.98 (3.92)	12.02 (3.74)		12.73 (3.81)		11.45 (3.52)		33.26		< 0.001		0.051	
Ego depletion	61.18 (9.07)	54.97 (9.69)		48.75 (9.25)		43.77 (9.88)		274.23		< 0.001		0.305	
Dropout intention	17.73 (6.38)	15.96 (6.06)		14.07 (6.03)		12.67 (5.87)		358.27		< 0.001		0.077	
ADHD symptoms	51.09 (6.03)	36.10 (4.54)		34.46 (4.73)		21.94 (10.86)		2439.13		< 0.001		0.796	

Note. Values represent means and standard deviations: M (SD) for each ADHD cluster.

The cluster effect was especially pronounced for ego depletion, maladaptive procrastination, and dropout intention. Medium-sized cluster effects were observed for maladaptive perfectionism and adaptive perfectionism. The effect of cluster on adaptive procrastination was smaller but still significant. Observed power exceeded 0.99 for all these effects, given the large sample size. In sum, the ADHD grouping (Combined, Inattentive, Hyperactive-Impulsive, Low) accounted for a meaningful proportion of variance in all six outcomes, with especially large effects for ego depletion and maladaptive procrastination, and a moderate effect for dropout intention. ADHD symptom severity differed strongly across clusters even after adjustment for age and biological sex. Consistent with this pattern, based on the ASRS clinical screening cut-off^37^, 315 students (92.9%) in the Combined cluster, 309 (56.2%) in the Inattentive cluster, 95 (20.2%) in the Hyperactive/Impulsive cluster, and 6 (1.2%) in the Low-symptom cluster scored above the threshold for clinically relevant ADHD symptoms. Among the 68 participants reporting a prior ADHD diagnosis, 34 (10.0%) were in the Combined cluster, 15 (2.7%) in the Inattentive cluster, 16 (3.4%) in the Hyperactive–Impulsive cluster, and 3 (0.6%) in the Low-symptom cluster.

### ADHD Cluster Differences on Procrastination and Perfectionism

Post hoc Bonferroni-corrected pairwise comparisons confirmed that the Combined type generally exhibited the most severe symptom profile, whereas the Low-symptom group showed the least severe profile, with the other two groups intermediate. For maladaptive procrastination, the Combined type reported the highest levels (M = 22.20) and the Low-symptom group the lowest (*M* = 14.80, 95% *CI* [6.30, 8.50], *p* < .001). All four clusters differed significantly from one another on maladaptive procrastination (*p* < .001 for each pairwise comparison). The Combined type scored about 2 points higher than the Inattentive type (mean difference = 2.00, 95% *CI* [1.30, 2.60], *p* < .001), approximately 6 points higher than the Hyperactive-Impulsive type (mean difference = 6.05, 95% *CI* [5.00, 7.20], *p* < .001), and about 7.4 points higher than the Low-symptom group (95% *CI* [6.30, 8.50]). Inattentive participants also had significantly higher maladaptive procrastination than Hyperactive-Impulsive peers (mean difference = 4.11, *p* < .001), who in turn scored higher than the Low-symptom group (mean difference = 1.33, *p* = .009). These results indicate a clear gradation, with greater ADHD symptom severity associated with more maladaptive procrastination.

A distinct pattern emerged for adaptive procrastination. The Low-symptom and Hyperactive-Impulsive groups reported the highest adaptive procrastination tendencies (*M* = 16.99 and 16.58, respectively), whereas the Combined and Inattentive types had the lowest (*M* = 14.60 and 14.96). The overall group effect was weaker (partial η² = 0.038). Post hoc tests showed that the Combined and Inattentive groups did not significantly differ from each other on adaptive procrastination (mean difference = 0.36, *p* = .993), nor did the Hyperactive-Impulsive vs. Low-symptom groups (mean difference = 0.41, *p* = .990). However, the Combined type was significantly lower in adaptive procrastination than both the Hyperactive-Impulsive (mean difference = − 1.98, 95% *CI* [–2.74, − 1.22], *p* < .001) and Low-symptom groups (mean difference = − 2.39, 95% *CI* [–3.13, − 1.62], *p* < .001). Similarly, the Inattentive type scored significantly lower than the Hyperactive-Impulsive (mean difference = − 1.62, 95% *CI* [–2.35, − 0.88], *p* < .001) and Low-symptom groups (mean difference = − 2.03, 95% *CI* [–2.74, − 1.28], *p* < .001). In other words, students in the high-symptom clusters (Combined and Inattentive) engaged in less adaptive delay than those in the Hyperactive-Impulsive or Low-symptom clusters.

For the perfectionism measures, the ADHD clusters showed a similar pattern. On maladaptive perfectionism, the Combined type had the highest mean score (*M* = 14.15) and the Low-symptom group the lowest (*M* = 11.71). The group effect was moderate. Post hoc comparisons indicated that the Combined type was significantly higher than all other groups (*p* < .001 in each comparison). The Low-symptom group was significantly lower than the Combined and Hyperactive-Impulsive groups (mean differences = − 2.43 and − 1.20, respectively, *p* < .001). The Hyperactive-Impulsive type scored slightly (but significantly) higher than the Inattentive type (mean difference = 0.71, 95% *CI* [0.16, 1.26], *p* = .012). The only non-significant pairwise difference was between the Inattentive and Low-symptom groups on this measure (mean difference = 0.49, 95% *CI* [–0.06, 1.08], *p* = .11), suggesting that those two groups had comparably lower levels of maladaptive perfectionism.

A nearly identical pattern was observed for adaptive perfectionism. The Combined cluster again showed the highest adaptive perfectionism (*M* = 13.95), significantly greater than all other groups (*p* < .001 in all cases). The Low-symptom group had the lowest adaptive perfectionism (*M* = 11.48), significantly lower than the Combined (*p* < .001) and Hyperactive-Impulsive groups (*p* < .001). The Hyperactive-Impulsive group (*M* = 12.70) scored significantly higher than the Inattentive group (*M* = 12.04; mean difference = 0.66, 95% CI [0.13, 1.17], *p* = .027). As with maladaptive perfectionism, the Inattentive vs. Low-symptom contrast was not statistically significant (mean difference = 0.55, 95% CI [–0.08, 1.19], *p* = .09). In summary, both perfectionism indices were elevated in the Combined-type ADHD group and lowest in those with minimal ADHD symptoms, with the Hyperactive-Impulsive subgroup tending to report slightly greater perfectionism than the Inattentive subgroup.

### ADHD Cluster Differences in Dropout Intention

ADHD clusters also differed significantly in reported dropout intention. The Combined type reported the highest dropout intention (*M* = 17.73), followed by the Inattentive (M = 15.96), Hyperactive-Impulsive (*M* = 14.07), and Low-symptom group (*M* = 12.67). All pairwise comparisons between clusters were statistically significant (*p* < .001), apart from the Inattentive vs. Hyperactive-Impulsive contrast (mean difference = 1.89, *p* = .065), which did not reach significance. The Combined group scored approximately 5.1 points higher than the Low-symptom group (95% *CI* [4.4, 5.7], *p* < .001), and about 3.7 points higher than the Hyperactive-Impulsive group (CI [3.0, 4.4], *p* < .001). The Inattentive group also scored significantly higher than the Low-symptom group (mean difference = 3.3, 95% *CI* [2.7, 4.0], *p* < .001). The effect of ADHD cluster on dropout intention was moderate in size.

### ADHD Cluster Differences in Ego Depletion

Finally, the groups differed markedly in ego depletion. The Combined type had the highest ego depletion scores (*M* = 61.18), followed by the Inattentive type (*M* = 54.97), Hyperactive-Impulsive type (*M* = 48.75), and then the Low-symptom group (*M* = 43.77). All pairwise comparisons between clusters on ego depletion were statistically significant (*p* < .001). In particular, the Combined type scored about 17.41 points higher than the Low-symptom group (mean difference = 17.41, 95% *CI* [15.48, 18.93], *p* < .001), roughly 12.44 points higher than the Hyperactive-Impulsive group (mean difference = 12.44, 95% *CI* [10.78, 14.13], *p* < .001), and about 6.20 points higher than the Inattentive group (mean difference = 6.20, 95% *CI* [4.67, 7.42], *p* < .001).

Likewise, the Inattentive group exceeded the Hyperactive-Impulsive group by 6.22 points (95% *CI* [5.03, 7.44], *p* < .001) and the Low-symptom group by 11.20 points (95% *CI* [9.96, 12.44], *p* < .001). Even the Hyperactive-Impulsive group had significantly higher ego depletion than the Low-symptom group (mean difference = 4.98, 95% *CI* [3.89, 6.07], *p* < .001).

This large separation among all four clusters on ego depletion corresponds to the largest effect size of the study (partial *η²* = 0.305 for the cluster effect on ego depletion).

### Effects of Age Group and Biological Sex

Controlling for ADHD symptoms, age group had no significant influence on any dependent variable. Neither the multivariate test nor any univariate test for age was significant (see Table 3), indicating that emerging adults (18–25 years) and young adults (26–35 years) did not differ in procrastination, perfectionism, ego depletion, or dropout intention scores when ADHD clustering and sex were accounted for.

In contrast, biological sex showed small but significant effects on several outcomes (see Table 2). There was no overall sex difference in adaptive procrastination (*p* = .060), but males reported slightly higher maladaptive procrastination (*M* = 18.5) compared to females (M = 17.8), a difference that reached significance (*F*(1, 1873) = 4.34, *p* = .037, partial η² = 0.002). Males tended to score lower on perfectionism than females: for maladaptive perfectionism, *F*(1, 1873) = 26.90, *p* < .001, partial η² = 0.014, and for adaptive perfectionism, *F*(1, 1873) = 44.15, *p* < .001, partial *η²* = 0.023.

**Table 3:** ANCOVA Summary with Age and Sex Effects.

Variable	Biological sex			Age		
	F	*p*	η²*p*	F	*p*	η²*p*
Maladaptive procrastination	*4.34*	*0.037*	*0.002*	*0.31*	*0.580*	*0.000*
Adaptive procrastination	*3.53*	*0.060*	*0.002*	*0.00*	*0.971*	*0.000*
Maladaptive perfectionism	*26.90*	< 0.001	*0.014*	*0.02*	*0.876*	*0.000*
Adaptive perfectionism	*44.15*	< 0.001	*0.023*	*0.03*	*0.857*	*0.000*
Ego depletion	*75.78*	< 0.001	*0.039*	*0.68*	*0.411*	*0.000*
Dropout intention	*6.81*	*0.009*	*0.004*	*0.19*	*0.662*	*0.000*

Note. Results reflect univariate ANCOVAs testing the effects of biological sex, and age group on six dependent variables. F-values, p-values, and partial eta squared (η²p) are reported for each variable.

In both cases, female students reported slightly more perfectionistic tendencies than male students (the adjusted mean differences between males and females were roughly 1 point on the perfectionism scales). Similarly, females exhibited higher levels of ego depletion compared to males (*F*(1, 1873) = 75.78, *p* < .001, partial *η²* = 0.039), with an average difference of about 5 points after controlling for ADHD cluster and age. Additionally, there was a small but significant effect of sex on dropout intention (*F*(1, 1873) = 6.81, *p* = .009, partial *η²* = 0.004), with female students showing slightly higher dropout intentions than males (*M* = 15.3 vs. 14.8). Although these sex differences were statistically significant due to the large sample size, the effect sizes were small.

## Discussion

The findings of this study contribute both theoretically and practically to our understanding of ADHD in university students. While past research has focused on inattentiveness as the primary academic risk factor^2^, our results point to the Combined-type cluster – marked by both inattention and hyperactivity – as the most compromised. One way to read the Combined cluster’s disadvantage is as a “double load” on self-regulation. When high inattention co-occurs with high hyperactivity–impulsivity, the student is not only fighting attentional drift but also contending with unstable impulse and arousal control. Dual-pathway^50^ accounts propose that ADHD-related impairment may reflect partly separable routes involving executive control dysregulation and delay-related motivational processes. Students showing elevations consistent with both routes may face combined strain on persistence and goal maintenance. In our data, this pattern appears as concurrent increases in maladaptive procrastination and subjective regulatory fatigue, alongside the highest dropout intention, a configuration compatible with a sequence from initiation difficulty to experienced self-regulatory cost and may in turn increase disengagement risk. This suggests that it is not merely the presence of symptoms, but their co-occurrence and intensity, that confer the greatest academic vulnerability. This pattern aligns with the dual-pathway model of ADHD^51^, in which impairments in both executive functioning and motivational regulation jointly contribute to functional difficulties. These findings also resonate with the cognitive-energetic model^52^, which posits that ADHD symptoms emerge from inefficient management of cognitive resources and arousal.

Not all delay, however, is dysfunctional. With respect to adaptive procrastination, students in the Combined-type cluster reported lower average levels than those in the Inattentive or Hyperactive-Impulsive clusters. This distinction matters: it can suggest that not all delay behaviors in ADHD are inherently bad, and that the context and style of procrastination determine its impact. This interpretation is conceptually consistent with multi-pathway (“triple pathway”) accounts of ADHD^51^, where delay-related processes are separable from other components, and may include both maladaptive (“delay-negative”) and potentially functional (“delay-positive”) patterns. In this light, active procrastination maps more closely onto a “delay-positive” profile (structured, deadline-contingent mobilization), whereas passive procrastination aligns with delay-related breakdown and accumulated self-regulatory cost. This pattern can imply that the strategic harness of heightened arousal from impending deadlines as a neurocognitive tactic to counter attentional deficits could be beneficial. The adaptive side of the pattern is less intuitive on first sight, but it becomes clearer if “delay” is treated as a strategy rather than a symptom. Active procrastination has been described as deliberate postponement paired with deadline-driven mobilization and a sense of control, often benefiting performance when time pressure amplifies focus^17^. The Hyperactive–Impulsive cluster’s higher active procrastination may reflect greater reliance on time pressure as an external regulator: the deadline supplies structure, elevates activation, and narrows behavioral options. By contrast, for the Inattentive and Combined clusters, time pressure may not translate into organized action as readily, because attentional instability can make the “last-minute sprint” harder to execute without fragmentation. Such a perspective enriches models of self-regulation in ADHD by incorporating the idea that certain “atypical” strategies can coexist with, or even compensate for, attentional deficits. Interventions might benefit from shifting away from pathologizing procrastination and instead teaching students to distinguish between passive and active forms. By recognizing when delay is purposeful, students may begin to reclaim agency and develop more adaptive ways of working under pressure.

A similar pattern emerged for perfectionism. This is consistent with prior research indicating that individuals with ADHD may exhibit predominantly maladaptive aspects of perfectionism – such as self-criticism when falling short of expectations – while showing less engagement with its adaptive components, like organization and the pursuit of high standards^10^. Students in the Combined-type cluster reported the highest levels of maladaptive perfectionism, while those in the Inattentive cluster did not differ meaningfully from the Low-symptom group. The elevation of perfectionism in the Combined cluster may reflect compensatory control attempts (higher standards and monitoring) that become costly when regulation is already strained, tipping toward self-criticism rather than effective organization^27^. This can suggest that perfectionistic distress is not a universal feature of ADHD symptomatology, but one that concentrates in individuals experiencing both attentional and hyperactive-impulsive symptoms. Interventions could therefore be selective, targeting perfectionism primarily in those with more complex symptom presentations, rather than applying broad assumptions across all ADHD subtypes.

Greater ADHD symptomatology was strongly associated with higher ego depletion; students with Combined-type ADHD symptoms report feeling most depleted, whereas those with minimal symptoms report the least ego fatigue. This aligns with the view that sustained self-regulation in the presence of both inattention and hyperactivity imposes a cumulative cognitive cost. Emerging research supports this conceptualization, demonstrating that ADHD students experience significantly higher daily cognitive exhaustion due to increased neural effort and diminished dopamine-mediated reward responses during prolonged cognitive tasks^53^. Recognizing this chronic depletion as micro-burnout underscores the necessity for targeted recovery strategies, such as structured cognitive breaks, mindfulness training, and adaptive energy management interventions, to ease cumulative academic fatigue and prevent attrition.

The inclusion of dropout intention as an outcome is also practically significant. Our results suggest a clear incline of academic risk: students in high-symptom clusters, especially those in the Combined group, are at elevated risk of dropout intentions compared to their peers with fewer symptoms. By demonstrating its links with ADHD symptomatology, similarly to prior research^54^ we highlight an early-warning signal that universities can monitor. If an increase in dropout contemplation is detected among students with high ADHD symptoms, it can trigger timely advisement or support outreach, potentially preventing actual dropouts.

### Clinical implications

Beyond theory, the present findings also speak to assessment practice: screening in higher education should not stop at symptom counts. The ASRS is a validated, symptom-focused screener anchored to DSM criteria and was not designed to quantify functional impairment or domain-specific academic dysfunction^37^. Given the centrality of impairment in adult ADHD assessment, a pragmatic next step could be to pair symptom screening with a brief, validated impairment measure suitable for college populations. If a strengths-inclusive snapshot is also desired, The Strengths and Weaknesses of ADHD Symptoms and Normal Behaviour Scale (SWAN;^55^ measures can be considered as an adjunct, although its use in adults should be guided by emerging adult psychometric work^56^ rather than assumed from child norms.

Beyond thresholds, there is also reason to consider whether the content and structure of adult ADHD assessment fully reflect developmentally salient manifestations in university settings^57^. Some authors have proposed potential emerging-adult symptom expressions that are not well captured by standard DSM symptom lists, including last-minute plan changes, procrastination-linked difficulties, low follow-through on commitments, emotional dysregulation, and fluctuating quality of work, while cautions that adding proposed adult symptoms may not necessarily improve diagnostic performance^58^. In this context, the ASRS item *“When you have a task that requires a lot of thought*,* how often do you avoid or delay getting started?”*^37^ captures delay broadly but does not distinguish strategic from maladaptive forms of procrastination. A theoretically useful refinement for future college-focused screening is to add a brief module that separates active from passive delay. For example, an active delay probe could read: *“I intentionally postpone starting academic tasks because I work most effectively under time pressure”* whereas a passive delay probe could read: “*I postpone starting tasks even when I want to begin and then struggle to initiate despite increasing consequences”.*

It may also be informative to assess perceived self-regulatory cost directly, given evidence linking depletion-related processes to academic procrastination in university students^12^. As an item from the measure of ego-depletion could capture “post-effort” self-control fatigue (e.g., *“After sustained mental effort*,* starting a new task feels substantially harder”*) alongside symptom screening. Collectively, these findings suggest that adult and college student screening may benefit from evaluating alternative symptom thresholds and models, and from adding brief, mechanism-relevant modules that improve functional specificity and guide intervention selection.

### Limitations

Several limitations of the current study should be acknowledged. First, the cross-sectional design limits our ability to draw causal inferences. It remains possible, for example, that students who are already experiencing academic failure or burnout might report heightened ADHD-like symptoms or specific coping behaviors as a consequence. Longitudinal research following students over time – from entry into university through potential withdrawal or graduation – would be invaluable to confirm the temporal ordering and causality of the relationships suggested by our findings.

Second, our reliance on self-report measures and an online survey methodology introduces concerns about report bias and measurement validity. Although we used well-validated instruments (e.g., the Adult ADHD Self-Report Scale)  and some evidence suggests that self-report measures may outperform implicit measures in capturing subjective psychological experiences^59^, it is important to note that elevated ADHD symptoms do not equate to a formal clinical diagnosis.

Additionally, the study’s sample demographics limit generalizability to a broader university population. We must acknowledge that many of our participants exhibited ADHD symptoms, with only a small percentage formally diagnosed with the disorder. Conversely, some students with genuine ADHD might under-report symptoms due to stigma or unawareness. As the ADHD diagnosis was self-reported and not clinically verified, and cluster assignment was symptom-based (ASRS) rather than diagnosis-based; clusters should be interpreted as profiles of current self-reported symptom presentation rather than clinical subtypes, which may limit generalizability to clinically confirmed samples and strengthens the case for future studies with diagnostic verification and information on treatment status. Future studies could strengthen this aspect by incorporating clinical evaluations or informant reports (e.g., from parents or clinicians) to validate group assignments.

Finally, we did not directly account for common co-occurring conditions such as anxiety, depression, or learning disorders. Comorbidity is the rule rather than the exception in adult ADHD^60^, and these co-occurring issues can independently contribute to academic difficulties.

## Conclusions

Our results show that the Combined-type ADHD symptom group experienced the highest ego depletion and maladaptive procrastination, indicating they may be at particular risk of mental fatigue and self-regulatory collapse during prolonged academic tasks. In contrast, the relative strength in adaptive procrastination observed in other ADHD symptom subgroups hints that these students might maintain performance by deliberately managing their time-pressure to stay engaged. By contributing this level of detail, our work helps move the field beyond one-size-fits-all approaches and towards personalized support strategies, thereby improving the academic success and well-being of students with ADHD symptoms.

## Data Availability

The datasets used and/or analyzed during the current study are available from the corresponding author on reasonable request.
